# Immune checkpoint inhibitor-induced myocarditis with myasthenia gravis overlap syndrome: A case report and literature review

**DOI:** 10.1097/MD.0000000000032240

**Published:** 2022-12-09

**Authors:** Loulu Gao, Xuemei Li, Zhijun Guo, Lin Tang, Jieqiong Peng, Bo Liu

**Affiliations:** a Department of Clinical Medicine, Weifang Medical University, Weifang, China; b Department of Oncology, Shandong Cancer Hospital and Institute, Shandong First Medical University and Shandong Academy of Medical Sciences, Jinan, China; c Department of Intensive Care Medicine, Shandong Cancer Hospital and Institute, Shandong First Medical University and Shandong Academy of Medical Sciences, Jinan, China; d School of Graduate, Shandong First Medical University and Shandong Academy of Medical Sciences, Jinan, China.

**Keywords:** immune checkpoint inhibitor, immune-related adverse events, myasthenia gravis, myocarditis

## Abstract

The therapeutic value of immune checkpoint inhibitors (ICIs) in a variety of tumors has been found and recognized, and although ICIs have improved the prognosis of many patients with advanced tumors, these drugs sometimes cause immune-related adverse events (irAEs).

**Patient concerns:**

We report a 67-year-old woman with advanced rectal endocrine tumor. Ten days after receiving two cycles of treatment with camrelizumab combined with http://www.baidu.com/link?url=shAWG4LYTwwBcZAEb6pLb6DkDndJR2tUgOfFiWAkOf0hS-_sj2jjSLBwYaxSiHY3r6yPj31Lp2DCP-7q3w7ho5HIV46V4fbIShFyUY7Cbka sorafenib, the patient suddenly suffered from chest tightness, shortness of breath and progressive aggravation of limb weakness, the high-sensitivity cardiac troponin T (hs-cTnT) was elevated to 3015pg/mL and N-terminal pro-B-type natriuretic peptide (NT-proBNP) up to 5671pg/mL, and creatine kinase (CK) was 1419U/L.

**Diagnosis and Interventions:**

The patient was diagnosed as immune checkpoint inhibitor-induced myocarditis with myasthenia gravis overlap syndrome. The patient was transferred to the intensive care unit (ICU) in time and given oxygen inhalation, glucocorticoids, immunoglobulin and anticholinesterase drugs, and other related treatments.

**Outcomes:**

After 2 weeks, the symptoms of myasthenia gravis (MG) were relieved, and the level of myocardial injury markers decreased significantly, but it was still at a high level. The patient's family refused further treatment, and the patient died soon after.

**Lessons:**

In this paper, Through the report and follow-up analysis of this case, this paper recognizes that the early correct understanding and evaluation of this fulminant and fatal irAEs and the reasonable treatment of patients are very important for the prognosis of patients.

## 1. Introduction

Immune checkpoint inhibitors (ICIs) are a major breakthrough in the history of cancer treatment, which benefits more and more patients. ICIs are monoclonal antibodies that restore the immune response of T cells to tumor tissues by blocking programmed cell death-1 (PD-1), programmed cell death-ligand 1 (PD-L1), and cytotoxic T-cell lymphocyte antigen-4. In the treatment of such drugs, it may cause immune-related adverse events (irAEs), such as enteritis, hepatitis, pneumonia, etc. These adverse reactions have a high incidence but are largely reversible. While the relative incidence rate of ICIs-related myocarditis is low, the mortality of patients is high, and some patients will also suffer from myasthenia gravis and myositis. Moreover, irAEs in many patients are often overlooked as tumor progression or paraneoplastic syndromes. In this article, we report a patient with severe irAEs after treatment with camrelizumab with sorafenib.

## 2. Case report

A 67-years-old woman reported that she had difficulty in defecation since November 2020, with occasional abdominal distension that was relieved by activity. She had a small amount of dark red blood in her stool intermittently. The patient had diabetes mellitus but denied having hypertension, hyperlipidemia, coronary heart disease and cerebrovascular disease. In March 2021, she came to the hospital and was finally diagnosed with a G3 grade rectal neuroendocrine tumor with multiple organ and lymph node metastases. Initially 5 cycles of octreotide acetate was given. In August 2021, she was admitted to the hospital for reexamination, and the results showed that the disease had progressed, so the treatment plan was changed to oral treatment with sorafenib. Since September 2021, 2 cycles of treatment have been carried out with camrelizumab on the basis of sorafenib.

When the patient returned to the hospital again for the next cycle, the patient reported difficulty in lifting the right eyelid and progressively aggravated limb weakness starting about 10 days after the end of the previous cycle. The patient suddenly felt chest tightness and shortness of breath 3 hours after admission. Physical examination revealed bilateral eyelid ptosis, especially the right eyelid. The patient was evaluated according to the Lovett muscle strength scale. The muscle strength of the patient’s right lower limb and left lower limb were grade IV and III respectively. Bedside electrocardiogram showed: sinus tachycardia (atrial rate 111 times/minutes), accidental atrial premature beats; multiple premature ventricular beats, short paroxysmal ventricular tachycardia, and sometimes ventricular fusion waves (mean ventricular rate 68 beats/minutes); ventricular escape; atrioventricular conduction abnormality; the ST-T segment changes. Through the examination of the patient’s hemodynamics, the results showed that the patient’s left ventricular systolic function was impaired. The high-sensitivity cardiac troponin T was 3015 pg/mL and N-terminal pro-B-type natriuretic peptide up to 5671 pg/mL, and creatine kinase was 1419 U/L. The white blood cell (WBC) and neutrophil (N) counts were high. IrAEs caused by camrelizumab treatment were considered and immediately transferred to intensive care unit for treatment (Table 1).

**Table 1:** Time of treatment and immune-related adverse events.

Time	Treatment and immune-related adverse events
2021/03–2021/07	**5 cycles of octreotide acetate**
2021/08	**1 cycle of sorafenib**
2021/09/09–2021/09/28	**2 cycles of camrelizumab plus sorafenib**
About 2021/10/08	**The patient developed muscle weakness and was not treated**
2021/10/13	**Camrelizumab-related myocarditis was diagnosed and transferred to ICU for treatment**

ICU = intensive care unit.

On the same day, the patient was given an intravenous injection of 360 mg methylprednisolone sodium succinate, followed by oral prednisone 50 mg, and then the methylprednisolone sodium succinate was reduced to 240 mg per day until the patient was discharged. During this period, patient was given oxygen inhalation, isosorbide nitrate, sodium creatine phosphate and other treatments were given to nourish and protect the myocardium, diuretics were used to reduce the cardiac load, and antiarrhythmic drugs were given, anticholinesterase drugs against muscle weakness. At the same time, anti-infection therapies, blood glucose control, correction of water, electrolyte and acid-base balance disorders and other treatments. On the third day of treatment, the symptoms of myasthenia gravis were slightly relieved. The patient had no obvious chest tightness and oxygen saturation was within the normal range. The patient’s family members refused to use immunoglobulin treatment at the beginning, but the patient’s cardiac function has not been significantly improved. From the fifth day, they were given immunoglobulin (25 mg/day) treatment for 6 consecutive days.

**Figure 1.**
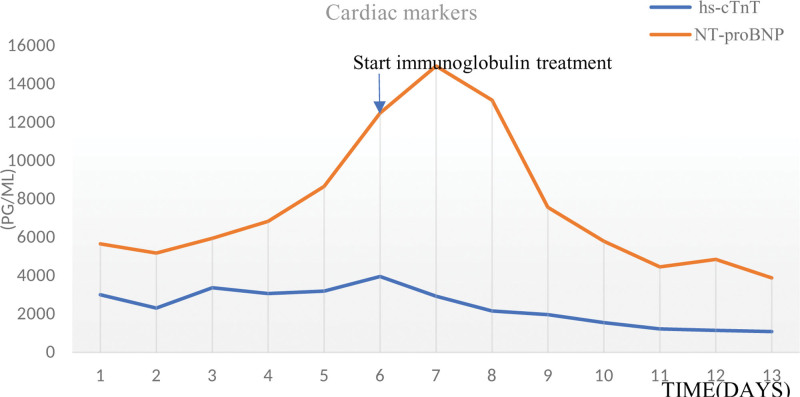
Changes of cardiac biomarkers in case.

On day 14, the high-sensitivity cardiac troponin T was 1099.00 pg/mL and N-terminal pro-B-type natriuretic peptide was 3891.00 pg/mL were significantly lower than before, but still at a high level (Fig.1), with no significant improvement in cardiac function, mild ptosis of the right eyelid, and muscle strength of both lower limbs reaching grade IV. The patient’s family refused further treatments for the patient and requested discharge. After follow-up, the patient was discharged without further treatment and eventually died.

## 3. Discussion

The rapid progression of irAEs reported in this paper has brought fatal consequences to patients. Therefore, comprehensive assessment of the risk of irAEs in patients as soon as possible will better improve the prognosis of patients. The etiological mechanism and risk factors related to irAEs are as follows:

Patients with their own underlying autoimmune diseases, cardiovascular diseases, diabetes and other risk factors may increase the risk of irAEs.^[1]^ In a study of patients with autoimmune cardiotoxicity, 5 out of 8 reported preexisting heart or vascular disease.^[2]^

More and more people believe that there are common antigens between the affected tissues and tumors in the adverse reactions of immunotherapy, leading to cross-reactions between tumor neoantigens and normal tissue antigens.^[3]^

The binding of immune checkpoints is necessary for T cells to maintain self-tolerance and regulate the duration and extent of peripheral tissue immune responses to mitigate peripheral tissue damage. The use of ICIs can lead to loss of self-tolerance by disrupting the balance between regulatory T cells and T effector cells, enhancing T cells effector function and leading to the development of fatal immune adverse reactions.^[4]^

Another mechanism is epitope spreading,^[5]^ tumor cell death leads to the release of large amounts of tumor antigens and autoantigens, which triggers an inflammatory cascade.^[6]^ It has been reported that ICI -associated myocarditis and myositis are mainly caused by damage due to infiltration of T lymphocytes with macrophages,^[7,8]^ and studies have shown that exosomes from PD-1 inhibitor-treated macrophages significantly promote cardiomyocyte senescence.^[9]^

ICIs are used in combination with several drugs that may increase the risk of cardiovascular disease, or have been used before ICIs treatment. For example, compared with patients treated with nivolumab alone, patients treated with nivolumab plus ipilimumab have an increased incidence and severity of myocarditis.^[10]^ Some anti-vascular endothelial growth factor treatments induce cardiotoxicity and are also associated with an increased risk of thrombosis and coronary ischemia.^[11]^

The patient was treated with sorafenib for 1 cycle, and then treated with sorafenib combined with camrelizumab. As a multi-target anti-tumor drug, sorafenib can act on tumor cells and block tumor angiogenesis by inhibiting vascular endothelial growth factor receptor and platelet-derived growth factor receptor. Therefore, it is speculated that the occurrence and progression of irAEs is exacerbated by the combination of sorafenib. Patient has diabetes, which also increases the risk of cardiovascular disease.

Before the last admission, the myasthenia symptoms of the patients we reported gradually worsened. The patients believed that it was the further development of the tumor and did not pay enough attention to it, which increased the difficulty of later treatment. Therefore, we need to improve the cognition of patients and their families treated with ICIs on the severity of irAEs, make a clear diagnosis as soon as possible and facilitate further treatment.

For patients with myasthenia gravis (MG) and myositis, they often feel fatigue and mild muscle soreness at the early stage with low specificity, and the early diagnosis is difficult and often ignored. The common clinical manifestations are ocular symptoms, gradual decrease of muscle strength in the limbs, leading to dyspnea and dysphagia in severe cases.^[12]^ The diagnosis can be assisted by detecting creatine kinase, electromyography, acetylcholine antibody titers, etc. Studies have reported that elevated acetylcholine antibody titers are not consistently associated with the development of immune checkpoint inhibitor-associated MG.^[13]^ Diagnosis can also be made by neurological examination, ice pack and endorphin tests, and serum tests.^[14]^ For the diagnosis of ICI-related myocarditis, although the specificity of myocardial injury markers, electrocardiogram and echocardiography is not strong, it is helpful to diagnose and evaluate the treatment effect. Troponin is more suitable than BNP as a marker of ICI-related myocarditis, because BNP in many cancer patients may be chronically elevated due to cancer-related inflammation and may be nonspecific.^[15]^

In order to accurately diagnose myocarditis, cardiac magnetic resonance imaging and, if necessary, endomyocardial biopsy can be performed.^[16]^ Among these, cardiac magnetic resonance imaging is the preferred noninvasive diagnostic test.^[17]^ It has also been suggested that the significant decrease of lymphocytes or the significant increase of neutrophils is associated with the prognosis of patients with ICI-related myocarditis.^[18]^

Timely transfer the patient to the intensive care unit, which facilitates real-time monitoring of all vital signs and continuous observation of changes in the condition, which needs to be advocated. Patients should first stop using ICIs and use high-dose steroids in time.^[19]^ If patients show signs of improvement within 24 hours, it is reported that the dosage of steroids will be gradually reduced within at least 4 weeks.^[15]^ For patients who do not respond immediately to high-dose steroids, intravenous Immunoglobulin treatment can also be given.^[20]^ At the same time, some immunosuppressive drugs can be given as adjuvant treatment, such as tacrolimus, infliximab, mycophenolate mofetil or anti-thymocyte globulin. It is worth noting that infliximab may be related to worsening heart failure, and it is forbidden to be used in patients with moderate to severe heart failure.^[21]^ For ICI-related myocarditis can also be supplemented with ACEIs, beta-blockers, diuretics, pacemaker placement, etc. If cardiogenic shock occurs, patients should be monitored in the cardiac intensive care setting and mechanical circulatory support can be applied.^[22]^ Some studies have shown that blocking OX40 and anti-4-1BB co-stimulation may also be an effective method.^[23]^ In addition, PD-1 inhibitors induce exosomes transfer of miR-34a-5p, leading to inhibition of PNUTS in cardiomyocytes, which leads to cardiac senescence. These findings may provide new targets for improving cardiac injury in immunotherapy patients.^[9]^ For this patient, the amount of glucocorticoid we gave was inadequate, and if immunoglobulin therapy had been given early, the patient would have improved faster and better.

In patients with MG, treatment with cholinesterase inhibitors is required, and some studies have shown that plasma exchange is more effective.^[24]^ In addition, it should be noted that many cases of ICI-related myasthenia crisis require ventilator support. Appropriate antibiotic treatment should also be considered to prevent or control infection.

IrAEs caused by ICIs should be paid attention to from many aspects. Before the use of ICIs, it is very important to comprehensively evaluate whether the patients have the risk factors for irAEs. Early diagnosis and treatment can improve the prognosis.

## Author contributions

**Data curation:** Loulu Gao, Xuemei Li.

**Formal analysis:** Zhijun Guo.

**Investigation:** Loulu Gao, Lin Tang, Jieqiong Peng.

**Writing – original draft:** Loulu Gao.

**Writing – review & editing:** Bo Liu.

## References

[1] Postow MA Sidlow R Hellmann MD. Immune-related adverse events associated with immune checkpoint blockade. N Engl J Med. 2018;378:158–68. 29320654 10.1056/NEJMra1703481

[2] Heinzerling L Ott PA Hodi FS. Cardiotoxicity associated with CTLA4 and PD1 blocking immunotherapy. J ImmunoTher Cancer. 2016;4:50. 27532025 10.1186/s40425-016-0152-y PMC4986340

[3] Johnson DB Balko JM. Biomarkers for immunotherapy toxicity: are cytokines the answer?. Clin Cancer Res. 2019;25:1452–4. 30587548 10.1158/1078-0432.CCR-18-3858 PMC6397678

[4] Kuhnly NM Coviello J. Immune checkpoint inhibitor-related myocarditis: recognition, surveillance, and management. Clin J Oncol Nurs. 2022;26:54–60. 35073300 10.1188/22.CJON.54-60

[5] June CH Warshauer JT Bluestone JA. Is autoimmunity the Achilles’ heel of cancer immunotherapy?. Nat Med. 2017;23:540–7. 28475571 10.1038/nm.4321

[6] Sury K Perazella MA Shirali AC. Cardiorenal complications of immune checkpoint inhibitors. Nat Rev Nephrol. 2018;14:571–88. 30013100 10.1038/s41581-018-0035-1

[7] Moslehi J Lichtman AH Sharpe AH. Immune checkpoint inhibitor-associated myocarditis: manifestations and mechanisms. J Clin Invest. 2021;131:e145186. 33645548 10.1172/JCI145186 PMC7919710

[8] Matas-García A Milisenda JC Selva-O’Callaghan A. Emerging PD-1 and PD-1L inhibitors-associated myopathy with a characteristic histopathological pattern. Autoimmun Rev. 2020;19:102455. 31838162 10.1016/j.autrev.2019.102455

[9] Xia W Chen H Chen D. PD-1 inhibitor inducing exosomal miR-34a-5p expression mediates the cross talk between cardiomyocyte and macrophage in immune checkpoint inhibitor-related cardiac dysfunction. J ImmunoTher Cancer. 2020;8:e001293. 33115945 10.1136/jitc-2020-001293 PMC7594538

[10] Shah RR Morganroth J. Update on cardiovascular safety of tyrosine kinase inhibitors: with a special focus on QT interval, left ventricular dysfunction and overall risk/benefit. Drug Saf. 2015;38:693–710. 26008987 10.1007/s40264-015-0300-1

[11] Liu Q Yu Y Lin J. Treatment strategy for myocarditis in patients using immune checkpoint inhibitors or combined anti-vascular endothelial growth factor therapy by clinical severity. Eur J Cancer. 2021;157:10–20. 34464781 10.1016/j.ejca.2021.07.023

[12] Safa H Johnson DH Trinh VA. Immune checkpoint inhibitor related myasthenia gravis: single center experience and systematic review of the literature. J ImmunoTher Cancer. 2019;7:319. 31753014 10.1186/s40425-019-0774-y PMC6868691

[13] Makarious D Horwood K Coward JIG. Myasthenia gravis: an emerging toxicity of immune checkpoint inhibitors. Eur J Cancer. 2017;82:128–36. 28666240 10.1016/j.ejca.2017.05.041

[14] Tedbirt B De Pontville M Branger P. Rechallenge of immune checkpoint inhibitor after pembrolizumab-induced myasthenia gravis. Eur J Cancer. 2019;113:72–4. 30986705 10.1016/j.ejca.2019.03.006

[15] Hu JR Florido R Lipson EJ. Cardiovascular toxicities associated with immune checkpoint inhibitors. Cardiovasc Res. 2019;115:854–68. 30715219 10.1093/cvr/cvz026 PMC6452314

[16] Zhou YW Zhu YJ Wang MN. Immune checkpoint inhibitor-associated cardiotoxicity: current understanding on its mechanism, diagnosis and management. Front Pharmacol. 2019;10:1350. 31849640 10.3389/fphar.2019.01350 PMC6897286

[17] Palaskas N Lopez-Mattei J Durand JB. Immune checkpoint inhibitor myocarditis: pathophysiological characteristics, diagnosis, and treatment. J Am Heart Assoc. 2020;9:e013757. 31960755 10.1161/JAHA.119.013757 PMC7033840

[18] Drobni ZD Zafar A Zubiri L. Decreased absolute lymphocyte count and increased neutrophil/lymphocyte ratio with immune checkpoint inhibitor-associated myocarditis. J Am Heart Assoc. 2020;9:e018306. 33190570 10.1161/JAHA.120.018306 PMC7763791

[19] Cautela J Zeriouh S Gaubert M. Intensified immunosuppressive therapy in patients with immune checkpoint inhibitor-induced myocarditis. J ImmunoTher Cancer. 2020;8:e001887. 33298621 10.1136/jitc-2020-001887 PMC7725077

[20] Caforio AL Pankuweit S Arbustini E. Current state of knowledge on aetiology, diagnosis, management, and therapy of myocarditis: a position statement of the European society of cardiology working group on myocardial and pericardial diseases. Eur Heart J. 2013;34:2636–48, 2648a. 23824828 10.1093/eurheartj/eht210

[21] Kwon HJ Coté TR Cuffe MS. Case reports of heart failure after therapy with a tumor necrosis factor antagonist. Ann Intern Med. 2003;138:807–11. 12755552 10.7326/0003-4819-138-10-200305200-00008

[22] Stein-Merlob AF Rothberg MV Holman P. Immunotherapy-associated cardiotoxicity of immune checkpoint inhibitors and chimeric antigen receptor T cell therapy: diagnostic and management challenges and strategies. Curr Cardiol Rep. 2021;23:11. 33483873 10.1007/s11886-021-01440-3 PMC7821837

[23] Simons KH de Jong A Jukema JW. T cell co-stimulation and co-inhibition in cardiovascular disease: a double-edged sword. Nat Rev Cardiol. 2019;16:325–43. 30770894 10.1038/s41569-019-0164-7

[24] Dhawan PS Goodman BP Harper CM. IVIG versus PLEX in the treatment of worsening myasthenia gravis: what is the evidence?. A critically appraised topic. Neurologist. 2015;19:145–8. 10.1097/NRL.0000000000000026 25970838

